# Assessing the effect of concerns about contraceptive-induced fertility impairment on hormonal contraceptive use by parity and residence: evidence from PMA Ethiopia 2020 cross-sectional survey

**DOI:** 10.1136/bmjopen-2023-077192

**Published:** 2024-08-13

**Authors:** Linnea A Zimmerman, Celia Karp, Solomon Shiferaw, Assefa Seme, Suzanne O Bell

**Affiliations:** 1 Department of Population, Family, and Reproductive Health, Johns Hopkins University Bloomberg School of Public Health, Baltimore, Maryland, USA; 2 Addis Ababa University, Addis Ababa, Oromia, Ethiopia

**Keywords:** REPRODUCTIVE MEDICINE, SEXUAL MEDICINE, PUBLIC HEALTH

## Abstract

**Abstract:**

**Objectives:**

This study aims to describe patterns of beliefs about contraceptive-induced infertility and assess their relationship with current contraceptive use, including whether these relationships vary by parity and residence.

**Design:**

We use data from Performance Monitoring for Action Ethiopia, a nationally representative, cross-sectional survey of 7491 women, aged 15–49, to assess agreement with the statement ‘If I use family planning, I may have trouble getting pregnant next time I want to.’ We used multilevel hierarchical models to identify the association between agreement and use of a hormonal method of contraception among 3882 sexually active, fecund women who wish to prevent pregnancy. We include interaction terms for parity and residence.

**Results:**

4 in 10 women disagreed (42.3%) and 2 in 10 strongly disagreed (20.7%) with the statement. Relative to women who strongly disagreed, women who disagreed and women who agreed had significantly lower odds of using a hormonal method of contraception (adjusted OR (aOR) 0.65, 95% CI 0.44 to 0.97 and 0.46, 95% CI 0.46, 95% CI 0.30 to 0.70). The effect of agreeing with the statement was strongest among high parity women (aOR 0.54, 95% CI 0.30 to 0.95). Greater agreement with the statement at the community-level use was associated with a reduction in the odds of using hormonal contraception but only among rural women.

**Conclusions:**

Efforts to address concerns around contraceptive-induced fertility impairment through the provision of comprehensive counselling and through community education or mass media campaigns are necessary, particularly among high-parity women and in rural communities. Interventions should acknowledge the possibility of delayed return to fertility for specific methods and attempt to address the root causes of concerns.

**STRENGTHS AND LIMITATIONS OF THIS STUDY**

- We use data from a unique survey item, derived from qualitative data in Ethiopia and other sub-Saharan African settings, that is not regularly available in other large-scale surveys.
- The large sample allowed for a sufficient sample size to explore relationships across both parity and residence.
- We do not include a specific time frame in the question which means we cannot differentiate between concerns about delayed return to fertility versus long-term or permanent impacts on fertility.
- We relied on the aggregation of individual beliefs from women of reproductive age; however, there may be many individuals in the community, including older women and men, who share this concern and who are not represented.
- We use cross-sectional data and cannot determine causality.

## Background

 Severe consequences exist for women who are unable to bear children, including adverse mental health outcomes, increased risk of intimate partner violence, abandonment, ostracisation and catastrophic financial repercussions.^1^,^4^ Fear regarding contraceptive-induced impacts on fertility is a critical barrier to use, particularly in sub-Saharan Africa.^5^,^8^ Existing evidence indicates no permanent impacts of contraceptive use on fecundity following discontinuation. Research does, however, suggest delays in return to fecundity for users of some hormonal methods—mainly injectable contraceptives—with delays of a subsequent pregnancy following discontinuation up to and exceeding 1 year.^9 10^ Concerns about contraceptive-induced fertility impairment, whether delayed fertility or permanent infertility, are so influential that they may preclude use of hormonal contraception altogether. Instead, women, particularly young women, may rely on either abortion^11^ or less effective methods of contraception, such as traditional methods or condoms, to regulate unwanted fertility.^12^ While using safe alternatives to hormonal contraception to regulate fertility is not inherently problematic, basing decisions on incomplete or inaccurate information is a reflection of reduced autonomy of individuals to make fully informed decisions; addressing these concerns is, thus, paramount.

Qualitative research from diverse settings has long described concerns related to use of hormonal contraceptive on subsequent fertility.^58 13^,^16^ Most studies do not distinguish between concerns about permanent sterility and delayed return to fertility, instead generally using ‘infertility’ to capture either permanent or temporary impacts on fertility, but are consistent in describing these concerns as a significant barrier to use of hormonal contraception and the non-hormonal intrauterine device (IUD). Recent quantitative research has highlighted that fear of contraceptive-induced fertility impairment, operating at both the individual level and among communities or social networks, can affect use. Social network research in Kenya found that the individual belief that contraception can cause infertility was associated with lower contraceptive use and that when members of an individual’s network also held this belief, contraceptive use was further reduced.^17^ Longitudinal evidence from Uganda found that women who strongly agreed that the use of contraception made it harder to get pregnant later were significantly less likely to adopt contraception over a 1-year period than women who did not hold this belief. There was no association, however, between higher levels of agreement within the community that contraception made it harder to get pregnant and an individual’s adoption of contraception.^18^ Odwe *et al* in Kenya found that concerns about long-term fertility impacts at the individual level were associated with method choice, but only among urban women. Though Odwe *et al* did not specifically explore community influences on use, the fact that results differed between urban and rural settings underscore that the influence of the social environment on contraceptive use is not uniform and can vary based on location and setting.^19^ Understanding how fears of contraceptive-induced fertility impairment operate at both the individual and community level can provide valuable information to guide responses to alleviate these concerns.

While studies suggest that the influence of fears regarding contraceptive-induced fertility impairment may vary based on environment, such as urban and rural residence,^19 20^ none have specifically explored whether the influence of contraceptive-induced fertility impairment might also vary over the life course, such as with increased age or parity. It is well established that increasing parity is linked to higher use of modern contraception, with consistently low percentages of nulliparous women using modern contraception across sub-Saharan Africa.^21 22^ Evidence suggests that fear of infertility is particularly pertinent for young women who have not yet finished, or even begun, childbearing,^5 11 15^ and this may, in part, explain low levels of contraceptive use among younger populations. Sedlander *et al* recently found that among Ethiopian female, rural, married, non-users of contraception, almost half (48.2%) believed that contraception caused infertility.^20^ They note that the importance of this concern is likely to vary by parity, given the social and familial pressures that women are under to demonstrate fertility on marriage^20 23^ but were unable to explore how concerns about contraceptive-induced fertility impairment varied by parity. Fears that long-term use will lead to infertility may be less concerning for women who wish to end childbearing, but few quantitative studies have explicitly explored whether the influence of fears related to contraceptive-induced infertility vary by age or parity level. A recent multicountry study by Bell *et al* found some evidence of greater perceptions of contraceptive-induced fertility impairment among women with fewer children in several geographies, but patterns by age were less consistent.^24^

Given the high level of injectable contraceptive use in Ethiopia (59% among married, modern contraceptive users)^25^ and research demonstrating the socially networked nature of beliefs about contraceptive-induced fertility impairment,^17^ understanding the ways in which such beliefs influence contraceptive decision-making may be particularly salient in Ethiopia. Our objective is thus to explore whether both individual-level and community-level beliefs of contraceptive-induced impacts on fertility are associated with contraceptive use and whether the effect of these beliefs varies by parity and residence.

We use data from Performance Monitoring for Action (PMA) Ethiopia, a nationally representative study of women aged 15–49, which captures women’s concerns about the effect of contraception on subsequent fertility. As the wording of the question, described in detail below, was not specific to permanent infertility, but rather concerns about difficulty in becoming pregnant in general, we will refer to this as a ‘fear of contraceptive-induced fertility impairment’, rather than fear of infertility, which we consider permanent sterility. First, we describe patterns of concerns about contraceptive-induced fertility impairment among all women and by sociodemographic characteristics. Second, we assess the relationship between these concerns and current contraceptive use, using both individual-level and community-level measures and assess whether these relationships vary by parity and residence. We hypothesise that women who agree that contraception adversely affects fertility are less likely to be using a hormonal method of contraception or the non-hormonal IUD. We also hypothesise that the effect of fears at both the individual and community level will vary by residence and parity, with the association between endorsing beliefs about contraceptive-induced fertility impairment and contraceptive use being stronger among low parity and rural women.

## Methods

### Data

We use data from the 2020 cross-sectional survey from PMA Ethiopia, a nationally representative data collection project. PMA Ethiopia employed a multistage sampling design to randomly select 231 enumeration areas (EAs) from all regions in the country, except for Tigray due to ongoing civil conflict at the time of the survey.^26^ All households in an EA were mapped and 35 were randomly selected. All women aged 15–49 who slept in the household the night before or who were usual members of the household were eligible to participate. Eligible women were approached by trained data collectors who explained study procedures and administered oral informed consent. All consents were provided as oral consent per guidance from the National Research Ethics Review Guidelines. Based on this guidance and from the IRB on record, written consent is not required in areas of low literacy or when data collection does not include invasive procedures (eg, biospecimen collection).^27^ Additionally, women aged 15–17 are considered able to consent from themselves for studies that cover sensitive topics, including reproductive health, and parental consent was not required per the National Research Ethics Review Guidelines. Additional details on study procedures, including a detailed study protocol, are published elsewhere.^26^ In total, 7533 de facto women completed the survey.

### Outcome measures

Our first objective is to describe the prevalence of concerns related to contraceptive-induced fertility impairment among all women. The primary variable of interest is agreement with the item, ‘If I use family planning, I may have trouble getting pregnant next time I want to’. This question was developed after extensive formative work during the PMA Women’s and Girl’s Empowerment in Sexual and Reproductive health (WGE-SRH) study, a multicountry, mixed-method investigation conducted in SSA.^28 29^ The first stage of the WGE-SRH study comprised qualitative in-depth interviews and focus group discussions with 320 women and men from Ethiopia, Nigeria and Uganda to explore themes related to the existence, exercise and achievement of reproductive choices for sex, pregnancy and contraception.^29 30^ Qualitative findings illustrated how women’s concerns about fertility, imbued with social pressures to conceive soon after marriage, informed decisions about contraceptive methods and use.^29^ The second stage of the WGE-SRH study translated qualitative themes into quantitative survey items that were evaluated as a sexual and reproductive health empowerment index.^28^ This analysis uses data from the item examining perceptions of contraceptive-induced fertility impairment, which was included in the survey without modification to wording. Initial qualitative work and pilot testing confirmed that in Ethiopia the term ‘family planning’ is largely synonymous with modern contraception, referring to use of technologies, rather than behaviours such as withdrawal and rhythm, to avoid pregnancy. Response options included strongly agree, agree, neither agree nor disagree, disagree and strongly disagree. ‘Do not know’ was not included as an answer option, though women could refuse to give a response if they were unable or unwilling to make a selection and were thus treated as missing and excluded from the final analysis (n=37). Respondents who were ambivalent could select ‘neither agree nor disagree’.

Our second outcome of interest is the current use of a hormonal method of contraception—specifically, implant, injectable, pill or emergency contraception—or the non-hormonal IUD. For simplicity, we refer to these as ‘use of a hormonal method of contraception’ and include non-hormonal IUD use in this category. Women were first asked whether they had heard of each method of contraception, including lactational amenorrhoea and traditional methods, and then asked if they or their partner were doing anything to prevent pregnancy. If so, they were asked to specify which method or methods they were using and were considered current users if they reported using any of the above-mentioned methods. Due to sample size limitations, we are unable to separate users of condoms (n=13) and traditional method users (n=127) as a separate group of non-hormonal contraceptive users and instead combine these with non-users of any method.

### Explanatory variables

In our descriptive analysis of fears of contraceptive-induced fertility impairment, we stratify by relevant sociodemographic characteristics, specifically; highest level of education attended (categorical variable; none, primary, secondary or higher), residence (urban/rural), marital status (currently married/unmarried) and fertility intention (wants more children, no more children, do not know, self-reported infertile) and wealth quintile. Wealth quintile was calculated using principal components analysis that included household items and household construction materials, following the same procedure as the Demographic and Health Surveys.^26 31^

In regression models, our primary explanatory variable is fear of contraceptive-induced fertility impairment. After exploratory analyses, we coded fear of contraceptive-induced fertility impairment into a three-level categorical variable, indicating (1) strongly agree/agree/neither agree nor disagree, (2) disagree and (3) strongly disagree as the relationship with use did not vary by strongly agree, agree and neither agree nor disagree but demonstrated a clear increase in association thereafter (online supplemental figure 1). For simplicity, we will refer to women who strongly agreed, agreed or neither agreed nor disagreed as ‘agree’. For consistency with the coding of the individual-level variable, community-level agreement was a continuous variable defined as the percentage of all women in the community who stated that they neither agreed nor disagreed, agreed or strongly agreed with the statement above (consistent with ‘agreement’ in the three-level categorical variable). Finally, we included parity, grouped as 0–1 to indicate low parity women, 2–3 to indicate middle parity women or 4+ to indicate high parity women. We conducted a sensitivity analysis separating women of parity zero and one. While effect sizes were largely similar, in the interaction models, statistical significance was attenuated, indicating insufficient power due to limited sample sizes of women of parity zero. We, thus, grouped women of parity zero and one in the final models.

### Adjustment variables

For the regression models, all explanatory variables described above were included with the exception of wealth and age. Wealth was highly correlated with residence (ρ=0.70), and age was highly correlated with parity (ρ=0.67). Given our focus on understanding residential and parity differences in our relationships of interest, we retained residence and parity in our model.

### Analytical sample

For descriptive analyses, we retained all women with non-missing values for the fear of contraceptive-induced fertility impairment and contraceptive use variables for a total of 7491 women (n=41 removed for missing values). This was also the sample used to calculate the community-level average. For regression analyses examining use of hormonal contraceptive methods, we excluded women who had never had sex, were currently pregnant, reported themselves to be infecund/menopausal, were users of female sterilisation or who wanted another child in less than 1 year as these would not be populations in need of contraception (n=3885). We conducted a complete case analysis and thus excluded three women who were missing responses to at least one adjustment variable for a total analytical sample size of 3882 from the original 7533 completed interviews.

### Analysis

We first used descriptive statistics (frequencies and proportions) to describe the sample and estimate the prevalence of fears of contraceptive-induced fertility impairment. All descriptive analyses accounted for clustering and differential probability of selection using design-based analysis and application of survey weights. Additional detail on survey weight construction is provided in the PMA Ethiopia protocol.^26^ We tested for differences across sociodemographic characteristics using the design-based F-statistic.

To explore the relationship between concerns about contraceptive-induced fertility impairment and contraceptive use, and whether the association between these concerns varied by residence and parity, we ran multilevel logistic regression models with EA as the second level and tested for interaction at multiple levels. As our central hypotheses were that the effect of concerns about contraceptive-induced fertility impairment varied by parity and residence, we tested for interaction between individual-level and community-level beliefs with both parity and residence and included those interaction terms that were significant (p<0.05) in at least one of the interaction combinations in the final model. Model 1 includes no interaction between parity and individual-level and community-level agreement and model 2 includes interaction between individual-level agreement and parity and community-level agreement and residence. To improve interpretability, we additionally used marginal effect models to estimate the probability of contraceptive use for model 2.

### Patient and public involvement

Patients or the public were not involved in the design, or conduct, or reporting, or dissemination plans of our research.

## Results

Table 1 shows sample characteristics for all women in the sample, used for descriptive analyses and of all sexually active, fecund women who wish to delay pregnancy for more than 1 year. Women in the full sample were younger, had lower parity, were more likely to want another child, be unmarried and have at least some education than women in the multivariable analytical sample. Opinions towards contraceptive-induced fertility impairment were similar, but women in the full sample were slightly more likely to agree or strongly agree with the statement. Across both samples, the average percentage of women in the community who agreed that contraception could affect fertility was about 36%. Approximately 7% of rural women lived in communities where between 75% and 100% of women agreed with the statement relative to fewer than 3% of urban women.

**Table 1:** Distribution of sample characteristics in full sample and analytical sample PMA Ethiopia 2020^25^

		Full sample		In need	
		N=7491		N=3882	
		n	%	n	%
Use of a hormonal method		1759	24.78	1662	43.66
User of a non-hormonal method		159	1.72	140	2.78
Age					
	Mean	-.-	27.93	-.-	30.47
Parity					
	0	2663	34.50	471	10.71
	1	1098	13.90	796	18.96
	2–3	1654	21.53	1148	29.19
	4+	2073	30.07	1467	41.14
Fertility intention					
	Wants more	5091	67.52	2383	62.41
	Want no more	1347	18.64	1210	31.05
	Do not know	831	11.47	289	6.54
	Reported infertile	211	2.37	-.-	-.-
Marital status					
	Not married	2734	34.72	766	16.55
	Married	4757	65.28	3116	83.45
Residence					
	Rural	4474	68.77	2396	70.78
	Urban	3017	31.23	1486	29.22
Wealth					
	Lowest	1279	19.66	626	19.45
	Lower	1267	18.78	707	19.89
	Middle	1289	19.45	725	20.61
	Higher	1380	18.92	746	18.72
	Highest	2276	23.20	1078	21.33
Education					
	None	2481	34.60	1518	42.33
	Primary	2742	39.09	1367	36.61
	Secondary+	2268	26.31	997	21.06
Contraceptive affects fertility					
	Strongly agree	479	6.45	174	4.71
	Agree	1848	24.78	862	22.32
	Neither agree/disagree	467	5.73	133	3.29
	Disagree	3401	42.35	1933	45.84
	Strongly disagree	1296	20.70	780	23.84
Community agreement		-.-	36.86	-.-	36.06

PMAperformance monitoring for action

**Figure 1:**
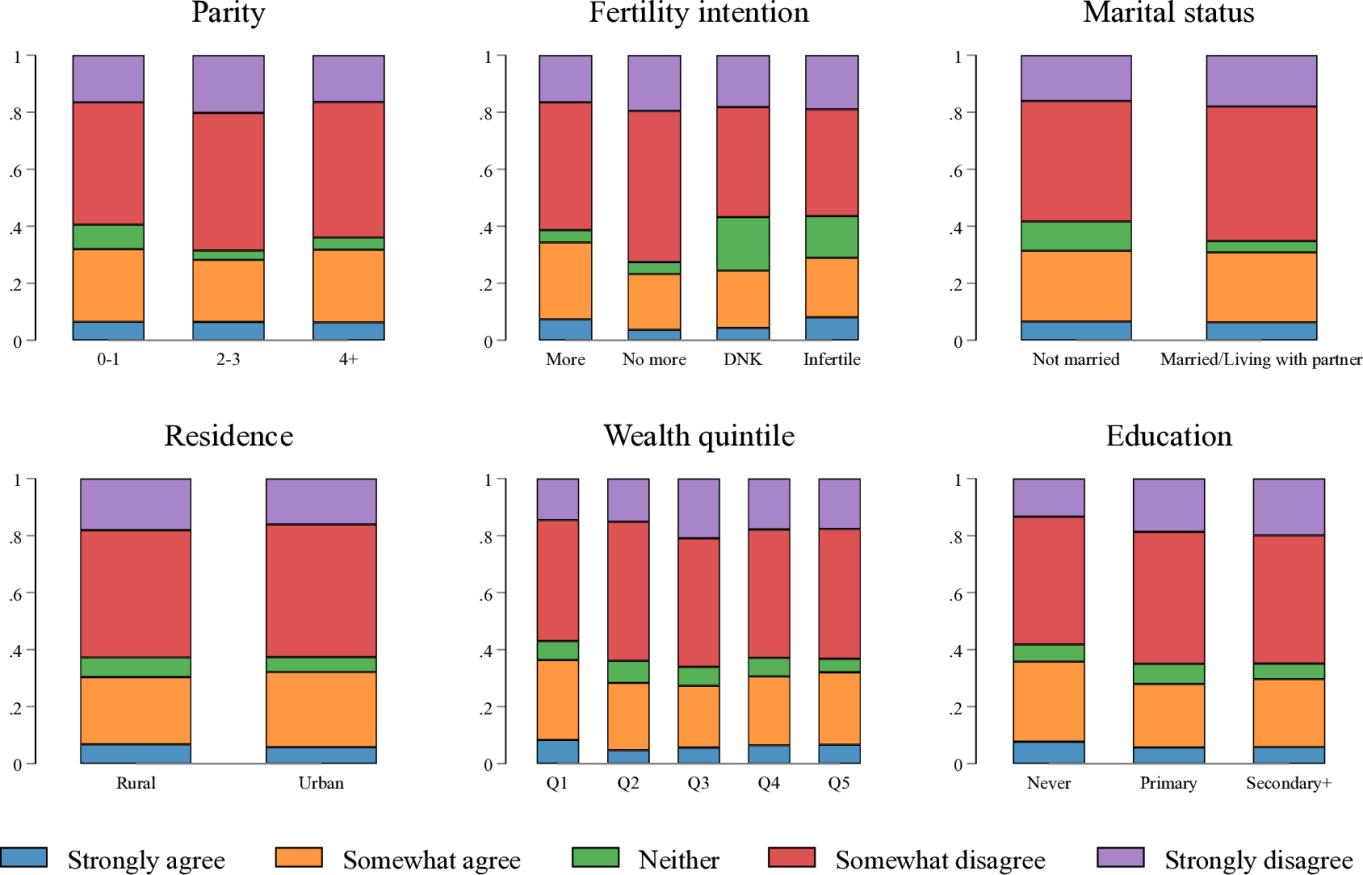
Agreement with statement ‘If I use family planning, I may have trouble getting pregnant next time I want to’ by sociodemographic characteristics.^25^

Figure 1 and online supplemental table 1 show the distribution of agreement with the statement that contraception may affect fertility by sociodemographic characteristics. There are significant differences across all sociodemographic characteristics, however, across all background characteristics more women stated that they disagreed with the statement—roughly 40%— than any other option.

Unadjusted results are shown in online supplemental table 2. Table 2 presents the results of the multilevel logistic regression, without interaction. Relative to women who strongly disagreed that use of contraception may affect fertility, women who disagreed and women who agreed had significantly reduced odds of using hormonal contraception (adjusted OR (aOR) 0.68, 95% CI 0.54 to 0.85 and aOR 0.39, 95% CI 0.30 to 0.50, respectively). At the community level, there was a significant negative association between the percentage of women in the community agreeing that contraception affects fertility and individual hormonal contraceptive use (aOR 0.14, 95% CI 0.06 to 0.33). Finally, parity was significantly related to hormonal contraceptive use, with increasing parity (2–3 children and 4+ children) related to a decline in hormonal contraceptive use (aOR 0.73, 95% CI 0.59 to 0.91 and aOR 0.45, 95% CI 0.35 to 0.59, respectively), relative to women with 0–1 child. The intraclass correlation coefficient (ICC) was 0.257 (not shown) indicating that approximately one-quarter of the variation in contraceptive use was due to variation across clusters. Supplemental analyses using disagree as the reference group confirm women who agreed with the statement were significantly less likely to use contraception than women who disagreed (aOR 0.57, 95% CI 0.47 to 0.70, not shown).

**Table 2:** Adjusted OR of hormonal contraceptive use among sexually active, fecund women wishing to delay pregnancy—without interaction; PMA Ethiopia 2020

		aOR	95% CI		P value
Agreement (ref: strongly disagree)					
	Disagree	0.68	0.54	0.85	<0.001
	Agree	0.39	0.30	0.50	<0.001
Community agreement		0.14	0.06	0.33	<0.001
Parity (ref: 0–1)					
	2–3	0.73	0.59	0.91	<0.001
	4+	0.45	0.35	0.59	<0.001
Residence (ref: urban)					
	Rural	0.49	0.35	0.70	<0.001
Fertility intention (ref: want more)					
	No more	0.85	0.69	1.04	0.12
	DNK/other	0.88	0.63	1.23	0.45
Marital status(ref: not married)					
	Married	9.75	7.56	12.56	<0.001
Education (ref: none)					
	Primary	1.41	1.15	1.73	<0.001
	Secondary and above	1.62	1.24	2.12	<0.001

PMAPerformance Monitoring for Action

**Figure 2:**
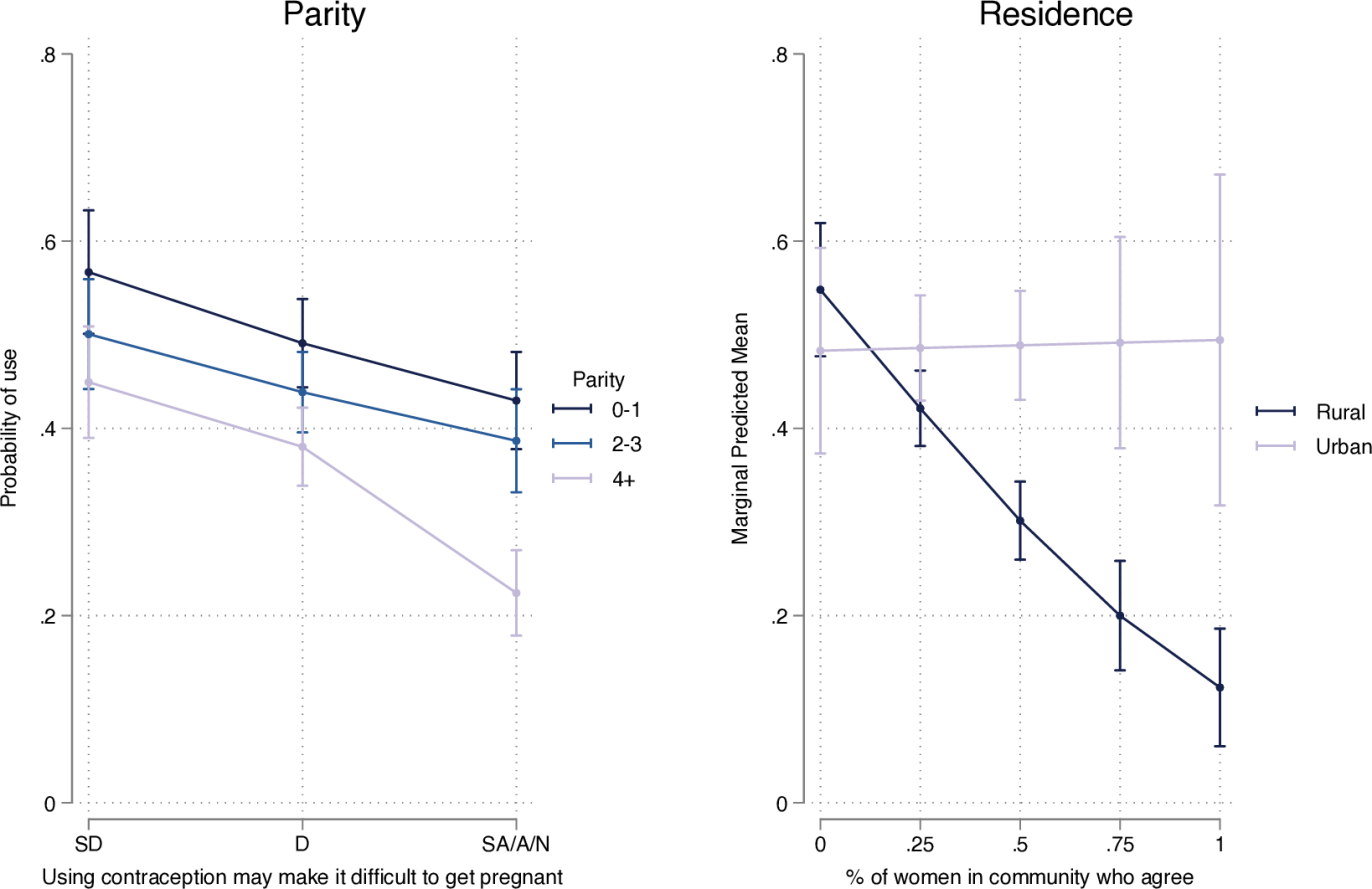
Predicted probability of hormonal contraceptive use by parity and residence; PMA Ethiopia 2020. PMA, performance monitoring for action.

Table 3 shows the results of the multilevel logistic regression, with interactions. On the whole, there is limited evidence of interaction by parity, except among high parity women for which any endorsement of beliefs of contraceptive-induced fertility impairment is associated with significantly decreased odds of using hormonal contraception relative to low-parity women. There is significant interaction at the community level, however. Among urban women, there is no association between greater agreement in the community and the odds of individual hormonal contraceptive use. Similarly, among women who live in a community where no women agreed that contraception use could impact fertility, there is no difference in the odds of individual hormonal contraceptive use between urban and rural women. Among rural women, however, greater community-level agreement with the statement about contraceptive-induced fertility impairment had a significant negative effect on individual hormonal contraceptive use. These relationships are visually shown in figure 2. All other relationships are generally consistent with the previous analysis, though there is no longer a difference in hormonal contraceptive use between women of parity 2–3 and 0–1. ICC estimates are consistent with the previous model at 0.250 (not shown). When compared with women who disagree, women who agree had reduced odds of using contraception (aOR 0.71, 95% CI 0.52 to 0.97, not shown) and significant differences in effect among the highest parity women remain (aOR 0.52, 95% CI 0.33 to 0.82, not shown). Online supplemental tables 3 and 4 show the results of the sensitivity analyses wherein parity zero and one were included separately.

**Table 3:** Adjusted OR of hormonal contraceptive use among sexually active, fecund women wishing to delay pregnancy—with interaction; PMA Ethiopia 2020

		aOR	95% CI		P value
Agreement (ref: strongly disagree)					
	Disagree	0.65	0.44	0.97	0.03
	Agree	0.46	0.30	0.70	<0.001
Parity (ref: 0–1)					
	2–3	0.69	0.44	1.07	0.10
	4+	0.51	0.32	0.82	0.01
Interaction term					
	2–3 and disagree	1.09	0.65	1.82	0.76
	4+ and disagree	1.04	0.62	1.73	0.89
	2–3 and agree	1.14	0.64	2.01	0.65
	4+ and agree	0.54	0.30	0.95	0.03
Community agreement		1.07	0.23	4.84	0.93
Residence (ref: urban)					
	Rural	1.44	0.69	3.02	0.33
Interaction term					
	Rural and EA agreement	0.05	0.01	0.34	<0.001
Fertility intention (ref: want more)					
	No more	0.85	0.69	1.04	0.12
	DNK/other	0.88	0.63	1.23	0.45
Marital status (ref: not married)					
	Married	9.75	7.57	12.56	<0.001
Education (ref: none)					
	Primary	1.40	1.14	1.73	<0.001
	Secondary and above	1.61	1.23	2.10	<0.001

PMAperformance monitoring for action

## Discussion

We find that concerns of contraceptive-induced fertility impairment are associated with lower hormonal contraceptive use in Ethiopia and that these concerns may be particularly influential for high parity women and among women who live in rural communities. We also found, however, that approximately 6 in 10 women either disagreed (42.3%) or strongly disagreed (20.7%) with the statement that using contraception would make it harder to get pregnant later, indicating that while these concerns are impactful, they may not be as widespread as qualitative evidence suggests.

We found that women who agreed that contraception could impact fertility were less likely to use contraception, aligning with recent research from Bell *et al* that found significantly reduced odds of contraceptive use among women who were concerned about contraceptive-induced fertility impairment in nine sub-Saharan African contexts.^24^ When examining whether the strength of this concern varied by parity, we found that concerns about contraceptive-induced fertility impairment were particularly impactful among high parity women, contrasting the largely qualitative literature that has highlighted the particular importance of this concern among young and nulliparous women.^5 6 11 12^ This is likely due in some part to reverse causality—women who are concerned about the safety of contraception are less likely to use it and thus, may be more likely to experience pregnancy. It does, however, suggest that high parity women may be a particularly important group to counsel on the safety of hormonal contraceptive methods, while acknowledging the potential for delays in return to fertility, as they are also more likely to wish to limit childbearing than young and low-parity women.^32 33^

Regardless of parity, it is clear that any level of agreement with this statement had a significant, negative effect on the probability of using contraception, underscoring that concerns about contraceptive-induced fertility impairment are a critical barrier to contraceptive use among women who wish to delay pregnancy. Unfortunately, we were unable to distinguish between whether women largely held short-term concerns, such as a delayed return to fertility, versus long-term concerns, such as permanent sterility and this is a critical question for future research. Delays in fertility, particularly among injectable users, have been documented. Acknowledging this possibility as part of counselling prior to adoption would serve to both validate the importance of women’s concerns and potentially alleviate later concerns should women experience a longer time to subsequent pregnancy than expected. As noted by Stevens *et al*, this information may be best presented through counselling strategies that identify the root cause of concerns and identify specific method-related beliefs so that women can choose the best method based on her personal beliefs and circumstances, rather than an explicit focus on correcting ‘mis’perceptions.^34^

After accounting for individual concerns of contraceptive-induced fertility impairment, we also found a strong association between higher levels of concern in the community and reduced probability of hormonal contraceptive use at the individual level, but only among rural women. Though not shown in our results, there was no difference in the association between individual-level agreement and hormonal contraceptive use between urban and rural women. The influence of individual-level concerns, thus, seems to operate largely the same between urban and rural women while community-level concerns affect contraceptive practices only among rural women. There are few studies to which we can directly compare our findings, given the dearth of quantitative research on the relationship between community-level beliefs of contraceptive-induced fertility impairment and individual contraceptive behaviour. A longitudinal study in Uganda found no effect of community-level beliefs related to contraceptive-induced fertility impairment on individual adoption, but findings were not stratified by urban vs rural residence.^18^ While not specific to modern contraceptive use at the individual level, Metheny and Stephenson demonstrated how norms related to fertility and family planning behaviour, when measured at the community level, differed in their effect on unmet need between urban and rural areas.^35^ The majority of other studies exploring the effect of social norms on family planning use tend to either be restricted to only urban^36 37^ or rural areas,^38 39^ or include residence as a confounding factor,^1840^,^42^ rather than an effect modifier. Nonetheless, our findings indicate that addressing more widely held concerns about contraceptive-induced fertility impairment may be particularly critical in rural areas. Programmes designed to engage communities and large-scale mass-media campaigns may be particularly pertinent means to address these beliefs. As in personal counselling, understanding the root causes of beliefs may also help to develop strategies that move beyond solely addressing beliefs about contraceptive use to addressing broader issues, such as the significant and severe consequences of infertility for women.

Our study is not without limitations, many of which are measurement related and would benefit from additional research efforts. We did not allow women to choose ‘do not know’ as a response option to the question related to contraceptive-induced fertility impairment, which has been demonstrated to be a relevant and predictive response for many fertility behaviours. However, we note that PMA surveys in Uganda and Kenya that used the same question wording found fewer than 3% of sexually active women chose ‘do not know’ as a response to this question. Though not directly comparable, this indicates that the vast majority of women in East Africa have formed some belief in relation to this statement. As noted in other studies^17 20^ and above, we do not include a specific time frame in the question which means we cannot differentiate between concerns about delayed return to fertility versus long-term or permanent impacts on fertility. Future research should attempt to differentiate between these concepts as they may differentially influence decision-making. Additionally, to estimate the influence of community-held concerns about contraceptive-induced fertility impairment, we relied on the aggregation of individual beliefs from women of reproductive age, however, there may be many individuals in the community, including older women and men, who share this concern and who are not represented. Finally, we asked about concerns related to contraceptive-induced fertility impairment arising from general contraceptive use, rather than identifying whether these concerns were associated with specific methods. Women may hold distinct beliefs about contraceptive-induced fertility impairment for specific methods versus others, which may shape their contraceptive behaviour in different ways. In addition to the measurement challenges outlined above, we use cross-sectional data and cannot determine causality. As noted above, this allows for the potential of reverse causality in our findings. Finally, given the inclusion of the question in a survey that largely focuses on family planning, it is possible that respondents felt some degree of social desirability to respond favourably to questions related to family planning and may have overstated their disagreement with the statement. Though we cannot confirm whether this bias is present, PMA Ethiopia has operated within the same or neighbouring communities since its inception in 2014 and is well regarded within communities, which may lead to greater trust with interviewers.

Despite these limitations, however, our study has a number of strengths. We use data from a unique survey item, derived from qualitative data in Ethiopia and other sub-Saharan African settings, that is not regularly available in other large-scale surveys to explore concerns in data that are nationally representative data among reproductive-aged women. The large sample allowed for a sufficient sample size to explore relationships across both parity and residence, adding further nuance to our understanding of how fears of contraceptive-induced fertility impairment operate. That data were nationally representative also improves generalisability to women in Ethiopia.

## Conclusion

We demonstrate the critical role that concerns about contraceptive-induced fertility impairment, held at both the individual and community level, play on contraceptive dynamics. While the majority of women in Ethiopia either disagree or strongly disagree that use of hormonal contraception may influence later fertility, efforts to address this concern, particularly among high-parity women and in rural communities, through the provision of comprehensive counselling and through community education or other mass media campaigns are necessary.

## supplementary material

10.1136/bmjopen-2023-077192 online supplemental file 1

10.1136/bmjopen-2023-077192 online supplemental file 2

## Data Availability

Data are available on reasonable request.
