# Supplementary figures and images for: Assessing the effect of concerns about contraceptive-induced fertility impairment on hormonal contraceptive use by parity and residence: evidence from PMA Ethiopia 2020 cross-sectional survey
Source: BMJ Open. 2024 Aug 13;14(8):e077192. doi: 10.1136/bmjopen-2023-077192 (PMC11331875; doi:10.1136/bmjopen-2023-077192)

Supplementary Figure 1: Predicted Probability of Contraceptive Use - Unadjusted

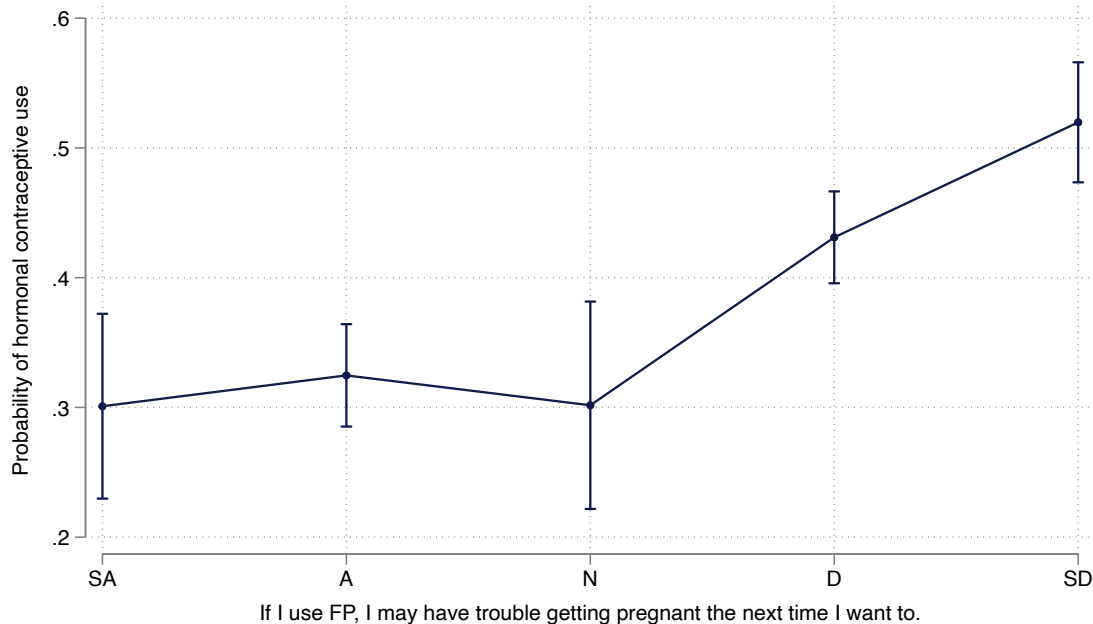

Supplement: online supplemental file 1 [file bmjopen-14-8-s001.pdf]
