# Supplementary material for: Assessing the effect of concerns about contraceptive-induced fertility impairment on hormonal contraceptive use by parity and residence: evidence from PMA Ethiopia 2020 cross-sectional survey
Source: BMJ Open. 2024 Aug 13;14(8):e077192. doi: 10.1136/bmjopen-2023-077192 (PMC11331875; doi:10.1136/bmjopen-2023-077192)
Supplement: online supplemental file 2 [file bmjopen-14-8-s002.pdf]

Table ST1: Agreement with statement ""If I use family planning, I may have trouble getting pregnant next time I want to" by sociodemographic characteristics (row percentages); PMA Ethiopia 2020

		Strongly agree	Agree	Neither agree or disagree	Disagree	Strongly disagree	p-value
Parity							
	0-1	6.2	26.1	8.0	40.1	19.5	0.000
	2-3	6.3	21.7	3.5	43.8	24.8	
	4+	6.9	24.8	3.7	44.9	19.7	
Fertility intention							
	Wants more	7.3	26.5	3.8	42.1	20.3	0.000
	Want no more	3.9	21.6	4.9	48.4	21.3	
	Do not know	4.5	21.1	16.5	34.8	23.1	
	Reported infertile	11.1	18.8	14.9	37.0	18.3	
Marital status							
	Not married	6.5	25.8	9.9	39.1	18.8	0.000
	Married	6.4	24.2	3.5	44.1	21.7	
Residence							
	Rural	6.6	23.6	5.9	42.0	21.9	0.000
	Urban	6.2	27.3	5.3	43.1	18.1	
Wealth							
	Lowest	5.9	26.0	5.4	43.3	19.4	0.000
	Lower	4.9	23.3	6.8	47.3	17.7	
	Middle	6.2	22.2	6.0	40.8	24.8	
	Higher	7.8	25.0	4.9	41.1	21.1	
	Highest	7.3	26.8	5.6	39.8	20.5	
Education							
	None	7.5	27.6	5.2	43.6	16.2	0.000
	Primary	5.7	22.4	6.2	42.9	22.8	
	Secondary +	6.3	24.5	5.7	40.0	23.5	

Table ST2: Unadjusted odds ratio of contraceptive use among sexually active, fecund women wishing to delay pregnancy

		aOR	95% CI		p-value
Agreement (ref: Strongly disagree)					
	Disagree	0.64	0.51	0.79	0.00
	Agree	0.35	0.28	0.44	0.00
Community agreement		0.16	0.07	0.36	0.00
Parity (ref: 0-1)					
	2-3	1.23	1.03	1.48	0.02
	4+	0.63	0.52	0.76	0.00
Residence (ref: Urban)					
	Rural	0.54	0.39	0.75	0.00
Fertility intention (ref: Want more)					
	No more	0.53	0.45	0.63	0.00
	DNK/ other	0.52	0.39	0.71	0.00
Marital status(ref: Not married)					
	Married	7.69	6.10	9.70	0.00
Education (ref: None)					
	Primary	1.83	1.53	2.19	0.00
	Secondary and above	2.18	1.76	2.71	0.00

Table ST3: Adjusted odds ratio of hormonal contraceptive use among sexually active, fecund women wishing to delay pregnancy – without interaction, reference category nulliparous women; PMA Ethiopia 2020

		aOR	95% CI		p-value
Agreement (ref: Strongly disagree)					
	Disagree	0.68	0.54	0.85	0.00
	Agree	0.38	0.30	0.49	0.00
Community agreement		0.14	0.06	0.33	0.00
Parity (ref: 0)					
	1	0.86	0.629	1.18	0.35
	2-3	0.65	0.47	0.90	0.01
	4+	0.40	0.28	0.57	0.00
Residence (ref: Urban)					
	Rural	0.51	0.36	0.72	0.00
Fertility intention (ref: Want more)					
	No more	0.86	0.70	1.06	0.16
	DNK/ other	0.83	0.59	1.16	0.27
Marital status(ref: Not married)					
	Married	10.75	8.17	14.14	0.00
Education (ref: None)					
	Primary	1.41	1.14	1.73	0.00
	Secondary and above	1.56	1.19	2.04	0.00

Table ST4: Adjusted odds ratio of hormonal contraceptive use among sexually active, fecund women wishing to delay pregnancy – with interaction, reference category nulliparous women; PMA Ethiopia 2020

		aOR	95% CI		p-value
Agreement (ref: Strongly disagree)					
	Disagree	1.05	0.55	2.04	0.88
	Agree	0.60	0.31	1.15	0.12
Parity (ref: 0)					
	1	1.34	0.67	2.69	0.41
	2-3	0.88	0.46	1.66	0.69
	4+	0.64	0.33	1.25	0.19
Interaction term					
	1 & Disagree	0.52	0.23	1.17	0.12
	2-3 & Disagree	0.64	0.31	1.35	0.24
	4+ & Disagree	0.63	0.30	1.31	0.22
	1 & Disagree	0.72	0.31	1.67	0.45
	2-3 & Agree	0.85	0.39	1.83	0.67
	4+ & Agree	0.40	0.19	0.88	0.02
Community agreement		1.16	0.26	5.28	0.85
Residence (ref: Urban)					
	Rural	1.54	0.73	3.22	0.25
Interaction term					
	Rural#EA agreement	0.05	0.01	0.31	0.00
Fertility intention (ref: Want more)					
	No more	0.85	0.69	1.05	0.14
	DNK/ other	0.82	0.58	1.15	0.25
Marital status (ref: Not married)					
	Married	10.59	8.05	13.93	0.00
Education (ref: None)					
	Primary	1.40	1.14	1.72	0.00
	Secondary and above	1.56	1.19	2.04	0.00
