# Supplementary material for: Assessing the effect of concerns about contraceptive-induced fertility impairment on hormonal contraceptive use by parity and residence: evidence from PMA Ethiopia 2020 cross-sectional survey
Source: BMJ Open. 2024 Aug 13;14(8):e077192. doi: 10.1136/bmjopen-2023-077192 (PMC11331875; doi:10.1136/bmjopen-2023-077192)
Supplement: Supplementary file 3 [file bmjopen-2023-077192.reviewer_comments.pdf]

PEER REVIEW HISTORY

BMJ Open publishes all reviews undertaken for accepted manuscripts. Reviewers are asked to complete a checklist review form and are provided with free text boxes to elaborate on their assessment. These free text comments are reproduced below.

ARTICLE DETAILS

**TITLE (PROVISIONAL)**	Assessing the effect of concerns about contraceptive-induced fertility impairment on hormonal contraceptive use by parity and residence; evidence from PMA Ethiopia 2020 cross-sectional survey
**AUTHORS**	Zimmerman, Linnea; Karp, Celia; Shiferaw, Solomon; Seme, Assefa; Bell, Suzanne O.

VERSION 1 - REVIEW

**REVIEWER NAME**	***Rose Stevens***
**REVIEWER AFFILIATION**	University of Oxford Social Sciences Division
**REVIEWER CONFLICT OF INTEREST**	I declare I have no competing interests
**DATE REVIEW RETURNED**	01-Aug-2023

**GENERAL COMMENTS**	Reviewer Comments to Author<br><br>Summary of the paper<br><br>As I understand it, this paper analyses a large and unique nationally representative dataset from Ethiopia on contraceptive use, that includes variables typically not included in other population health surveys – including their question of interest: agreement with the statement "If I use family planning, I may have trouble getting pregnant next time I want to". The analysis is in two parts: 1) a descriptive summary of agreement with this statement across various demographics and 2) an examination of the association between agreement with the statement (at the individual and community level) and current hormonal contraceptive use. The latter is explored in interaction with parity and urban rural residence. The results show that agreement with the statement is associated with a lower odds of contraceptive use, particularly among high parity women. Higher community level agreement is also associated with reduced odds of use among rural women only.<br><br>Overview of recommendation and major comments<br><br>Recommendation: Accept with minor revisions<br><br>Strengths: I think that the focus of this paper, the statement in question, is a unique survey item and assessing beliefs about and the experience of physiological impacts of contraceptive use is an important and timely topic. Understanding this in the context of variation in parity and residence is also novel and insightful. I think having a descriptive picture of who agrees with this belief in such a large, representative data source and across demographic groups is a huge contribution in and of itself. If there's space, I'd love to see this section bumped up with a visualisation of some kind if the authors think that would be of value. Showing the expected

	association between agreement with this statement and contraceptive use in this dataset adds to the evidence base on this topic and would merit being published.<br><br>Limitations: The authors identify well that there is ambiguity in the belief statement as to whether the worry is about delayed return to fertility or long-term permanent impacts on fertility and that this requires future research to pick apart. Whilst the question wording cannot be changed at this point, I think that the conclusions and implications of this manuscript should be tempered to account for this ambiguity.<br><br>It would be good to see the different 'infertility' terms and their meanings carefully set out to start with, with reference to what the authors think the question statement is mostly being interpreted as meaning by respondents (as far as can be speculated). I am of the view that for some women, concerns about post-use infertility and subsequent non-use of contraception may be understandable, and in some senses, rational given the context they find themselves in, particularly given the huge implications of infertility in Ethiopia, as they authors identify. It would be good to see consideration of the possibility that some of these women may be acting in their best interests and that we should not automatically write them off as 'wrong' (see Stevens et al 2023). As I mention in comments below, research does show there is lots of variation in return to fertility time among contraceptive users, particularly injectable users. Additionally, studies of fertility decisions among women in more uncertain economic circumstances in LMICs show that they are less forward planned and more about flexibly timing their fertility to become pregnant when a safe or suitable time arises, and that contraceptives with uncertain return to fertility times can foreclose possibilities to have a child when the time arises (see Johnson-Hanks 2015 and Trinitapoli and Yeatman 2018).<br><br>Given this, I question whether we should be counselling women that agreeing with the statement at the centre of this paper is wholly wrong, and that whether that does indeed give women autonomy over controlling the fertility in the way they'd wish. Even if the statement were 'wholly wrong', recommendations to counsel women out of holding these beliefs has limited evidence for its efficacy anyway (see Cavallaro 2020).<br><br>I think the results of this paper are interesting and fairly robust. But I am not convinced that the conclusions and implications directly follow from the results, and would like to see a bit more space in the introduction and discussion (plus abstract conclusions) to account for the assumptions and nuances behind the statement of interest.<br><br>Finally, the other two main comments I have, which are elaborated on below, are:<br>- I would like to see how the parity 0 group differs from parity 1.<br>- I would like to see how many individuals answered don't know to the statement and whether they were included in the analysis. If so, which group were they in? Don't know responses in surveys can indicate inattentiveness or worry of judgement etc. and could actually be quite informative (See Alvarez et al 2019).<br><br>Refs

	Alvarez, RM. et al. (2019) 'Paying Attention to Inattentive Survey Respondents.' Polit Anal. 27, pp145-162. doi:10.1017/pan.2018.57<br><br>Cavallaro, F.L. et al. (2020) 'A systematic review of the effectiveness of counselling strategies for modern contraceptive methods: what works and what doesn't?', BMJ sexual & reproductive health, 46(4), pp. 254–269. Available at: https://doi.org/10.1136/BMJSRH-2019-200377.<br>Johnson-Hanks, J. (2015) 'When the Future Decides', Current Anthropology, 46(3), pp. 363–385. Available at: https://doi.org/10.1086/428799.<br>Stevens, R. et al. (2023) 'Misconceptions, Misinformation, and Misperceptions: A Case for Removing the "Mis-" When Discussing Contraceptive Beliefs', Studies in Family Planning, 54(1), pp. 309–321. Available at: https://doi.org/10.1111/sifp.12232.<br>Trinitapoli, J. and Yeatman, S. (2018) 'The Flexibility of Fertility Preferences in a Context of Uncertainty', Population and Development Review, 44(1), p. 87. Available at: https://doi.org/10.1111/PADR.12114.<br><br><br>Minor comments<br><br>Key words<br><br>1. I would consider switching 'infertility' to 'fertility impairment' as per the title or adding 'delay in fertility' in as well to handle the ambiguity in the statement.<br><br>Background<br><br>2. I really like the first sentence. Acknowledging the impact of infertility on women and the lack of services for women in LMICs is central to this topic.<br><br>3. Line 72-75 – it would be useful to have some indication of how long this short delay is based on existing research. I feel the reader should be able to assess themselves what current evidence shows on differences in fertility delays across methods.<br><br>e.g. The discussion of the below paper is quite interesting as a discussion of return to ovulation following different dosages/regimens/trial conditions of the injectable, which is of course, the most commonly used method in Ethiopia.<br><br>Taylor DJ, Deese J, Bahamondes L, Brache V, Veiga N Jr, Fuchs R, Halpern V, Dorflinger LJ. Return to ovulation after Sayana Press is injected every 4 months for one year: Empirical and pharmacokinetic/pharmacodynamic modeling results. Contracept X. 2022 Jul 25;4:100080. doi: 10.1016/j.conx.2022.100080. PMID: 35965654; PMCID: PMC9372597.<br><br>4. Line 79 – 82 – it's great to see a discussion of contraceptive autonomy brought in, and I do agree that full, supportive and high-quality information is a part of contraceptive autonomy. What the 'right' information to give on fertility delays post-contraceptive use is more complicated.<br>5. I would love to see a brief definition somewhere in the background of 'infertility' and 'fertility impairment' or delay or any other terms used etc. so we know what we are referring to with

	each. What's the difference? Does one imply permanence or longer duration etc.<br>6. Line 106 – 108 – great to see discussion of fear of infertility driving low use among women with no children. Good justification for the need to investigate this question in interaction with parity.<br><br>Methods<br><br>7. Line 160 – 161 - Re the statement of interest: "If I use family planning, I may have trouble getting pregnant next time I want to" – Were women at any point during the survey told that they should be thinking about modern methods of contraception as opposed to say lactational amenhorrea, or was this not specified? Perhaps mentioning the assumptions behind what family planning meant to individuals taking the survey would be useful. Great to see that the question was developed based on prior mixed methods research and to summarise that research briefly here – it strengthens the question in my eyes.<br>8. Line 176 – 178 – good to have a rationale for the language used here. Perhaps this is a bit that could be detailed further to include definitions and assumptions of the different infertility related terms used in the study and the background as recommended above.<br>9. Line 190 – could you add a little detail on how wealth quintile was calculated (or cite the protocol maybe if it's in there).<br>10. Line 194-195 – What did you do with the don't knows? Don't knows can be an important group. They may be worried about social desirability for instance and thus may be concealing an 'agree' answer. It would be worth understanding who this group are and maybe including them in the analysis.<br>11. Line 202 – 204 – Personally, I would consider having 0 parity women grouped separately. As a lot of fear about post - use infertility may be to do with proving your fertility once your married and having that first child, I would imagine different associations between those with no children and those with any. Does it ruin your power if you split out the 0 children individuals? Perhaps adding a supplementary sensitivity analysis to see how 0 and 1 parity women differ would be useful if you don't want to change the full analysis. Or even run an analysis with any children vs no children. I'd love to see a line to tell us if this makes a difference to the results even if you don't include any extra analysis.<br>12. Line 219 – Do you have information on if women are currently using other traditional or non-hormonal methods of contraception, such as lactational amenhorrea, condoms, or withdrawal either as their sole method or in combination with a hormonal method? It would be good to see descriptive statistics on this somewhere and a consideration of whether agreement with this belief is driving 'non-use' or 'use of non-hormonal methods'.<br>13. Line 223 – 238 – Analysis methods look good to me. It would be great to see in the results of the multilevel models which levels (at the EA level or at the individual level) accounted for what proportion of the variation in the outcome. Was there more variation in contraceptive use at the level of the EA or of the individual? Additionally, you state that "We tested for interaction between individual- and community-level beliefs with both parity and residence and included only those interaction terms that were significant in the final model". Please outline how you determine 'significance' of the interaction term? It seems you only required one of the interaction combinations to show significance to retain it. I'm slightly concerned about whether this shows enough evidence for an interaction and warrants keeping this interactive effect in the model.

	It is true that the 4+ / agree group shows an interesting finding, but it would be great to have a clear justification, back up for why you keep that interaction included.<br><br>Results<br><br>14. Line 251 – What do you mean by negative views?<br>15. Table 1 - Could you also include descriptive statistics on whether individuals were breastfeeding exclusively, not exclusively, not at all. Would be good to consider from a contraceptive ‘need’ perspective.<br>16. Table 2 – Please report % don’t knows as well. Again, I would like to see parity split into 0 and 1. Just a thought, and I don’t know what your limits are on words/figures, but I think some figure/visualisations would be really helpful with these descriptive statistics. It’s quite hard to see the trends just with a table, although still incredibly useful. A few relative frequency plots would illustrate some of the trends nicely and even allow two way comparisons (e.g. show agreement across parity levels split by urban rural residence, or show agreement across education levels split by marital status). It think it would be a nice aide to any researchers trying to get a good picture of which groups of women agree with this statement. This isn’t crucial, but would add to it somewhat I think.<br>17. Table 3 – EA agreement slightly confused me. Could you change it to community agreement? Also, it’s interesting that fertility desires are not associated with contraceptive use – could you comment on that if you have space (just out of interest as opposed to necessary). Do you think it’s due to selecting down to only women ‘in need’?<br>18. Figure 1 is very useful and compelling on the community type interaction. I am left wondering how many urban and rural communities had the different quartiles of agreement level. Is there any way you can show this on the figure or talk about it descriptively e.g. how many urban communities actually had 75% - 100% agreement with the statement for instance.<br>19. Table 4 and generally – is there evidence in each regression analysis, if you change the reference category for the main exposure, for a difference between the disagree and agree responses? Maybe just adding one sentence on this somewhere would be useful.<br><br>Discussion<br><br>20. The summary of the results in the first line of the discussion is a little confusing to me. It would help me if you differentiated the results for community level concerns and individual level concerns.<br>21. Line 327 – 328 – Do you find this in your data that higher parity women are more likely to want to limit childbearing? Is there are a greater proportion of 4+ parity women who say they want no more children than 2-3 parity women say?<br>22. Lines 362-380 - Limitations – good to acknowledge that these effects may be method specific. I would expect them to differ by method. Also good to add information about the risk of social desirability bias. Can you say any more on this? Who were the interviewers used, is there qualitative work you could draw upon to understand what why they might consider ‘disagreeing’ desirable or what pressures/normative sanctions they faced?<br>23. Lines 382 – 389 – Very strong strengths as a dataset and progressive to have included the statement of interest.

	General<br><br>24. Do you have any data on past use of contraception? You could look at method specific past use (e.g. have they used the injectable in the past vs the implant vs none) and see if that impacted belief. This would reduce some of the cross-sectionality issues and would be interesting. Perhaps for a different paper!

**REVIEWER NAME**	***Elvis Junior Dun-Dery***
**REVIEWER AFFILIATION**	Oregon State University, Public health and Nutrition
**REVIEWER CONFLICT OF INTEREST**	I have no competing interest
**DATE REVIEW RETURNED**	25-Sep-2023

**GENERAL COMMENTS**	Review report – bmjopen-2023-077192<br>Topic: Assessing the effect of concerns about contraceptive induced fertility impairment on hormonal contraceptive use by parity and residence; evidence from PMA Ethiopia 2020 cross-sectional survey.<br><br>Abstract:<br>Comment 1: Abbreviations are first defined before subsequent abbreviations. Please refer to PMA in topic and objectives.<br>Results:<br>Line 1: Six in ten will not be calculated as 42.3%. Please check this and it may be 60%.<br>Line 1: If disagreed and strongly disagreed are different "variables", then "or" should be replaced with "and".<br>Discussion: Please replace discussion with a conclusion. Also check "………other mass media campaigns are necessary are critical,". Did the authors want to say, "necessary and critical".<br><br>Strengths and limitations of the study<br>Comment: Please add a sentence or two about what new knowledge the study adds to existing knowledge and available literature. Then reduce the length of this section.<br><br><br>Main document<br>Background:<br>Comment 1: Line 75 - 79…Consider reducing the length of sentences. That gives a better meaning to readers.<br>Comment 2: Authors may want to explain what they mean by " …… amplified when network members also held this belief…."<br>Comment 3: line 90: …… strongly agreed that the use of contraception…."<br>Line 91 – 92: …… were significantly less likely to adopt contraception over a one-year period;<br>there was no association, however, between levels of community agreement and uptake". Authors may want to explain this better. The two sentences really are not connected.<br>Comment 4, line 96 - 97: that results differed between urban and rural settings speak to the diverse ways in which this concern and the social environment can influence contraceptive use. Please rephrase this sentence clearly.<br>General comment: Authors may want to consider adding more data to demonstrate broader evidence of the subject in Sub-Saharan Africa. Also compare statistical evidence between Ethiopia and

	other African countries, not just Kanya. Ensure that sentences and paragraphs are connected in meaning and content.<br><br>Methods, Data:<br>Comment 1,: Please state specifically the multistage sampling method that was used by PMA or give a reference for readers.<br>Comment 2: Multilevel hierarchical models assessed how agreement is associated with the use of a hormonal method of contraception among 3,882 and descriptive statistics used among a sample of 7,491 women. In total, 7,533 de facto women completed the survey (refer to main body)? Can authors explain if the 3,882 sample is part of the 7,491 women? Refer to abstract and methods in main document. Also reconcile the 7,533 sample with the rest. Please make clear as it is under the heading “Analytic sample” but reconcile with 7,533 sample.<br>Include a sentence about ethical consideration for women less than 18 years of age. Were they also under oral consent and by parents or not?<br><br>Outcome Measures:<br>Comment 1: How did authors measure the second outcome “current use of a hormonal method of contraception”?<br>Line 190: Was “reported infertility” viewed by authors as self-reported or considered as contraceptive-induced?<br>Line 197 -198: If authors categorized “neither agreed nor disagreed” as agree, was this analyzed as a separate variable or combined with “strongly agreed or agreed” as a single variable? I would have thought that this would be an independent/neutral group. Authors previously stated in line 175 that “respondents who were ambivalent were categorized as “neither agree nor disagree”, then further categorize this as agree.<br>Define your terms as they relate to your study variables for a wider audience. E.g: contraceptive-induced infertility, multilevel hierarchical models, parity nulliparous and primiparous, etc.<br><br>Results<br>Comment 1: Authors may want to explain (in a single sentence) the concept of full sample and analytic sample in the methods section before incorporating it here.<br>Line 261-262: 40% is less than the other half (60%) and cannot be considered as the larger percentage.<br>Line 292: “…. among women who live in a community where no women agreed...”. Did authors want to mean no woman or no women?<br>Authors should give brief descriptions of figures.<br>General comment: Please move all table headings to where tables are located or delete table headings from results section if tables below already contain headings. Only reference text descriptions in the results corresponding to tables.<br><br>Discussion<br>Comment 1, line 312: please refer to comment on the abstract.<br>Comment 1: Authors may want to reduce the length of sentences to make meaning.<br>Comment 1: Authors may want to compare results to more findings than they already did. More is needed on which other findings by other authors are different and which are like current findings?<br><br>References

	Comment 1: Authors may want to review old references and update with recent ones.

VERSION 1 – AUTHOR RESPONSE

We thank the editor for the opportunity to revise and resubmit following our original oversight in not responding to Reviewer 2's comments. We have revised the manuscript per their suggestions and believe it has improved the readability of the manuscript. Please note that we incorporated these edits into the original response to Reviewer 1 so that all changes based on reviewer comments were consolidated in one draft.

Review report – bmjopen-2023-077192

Topic: Assessing the effect of concerns about contraceptive induced fertility impairment on hormonal contraceptive use by parity and residence; evidence from PMA Ethiopia 2020 cross- sectional survey.

Abstract:

Comment 1: Abbreviations are first defined before subsequent abbreviations. Please refer to PMA in topic and objectives.

Response: Thank you for identifying this. We have defined PMA as Performance Monitoring for Action (PMA) when we first refer to it.

Results:

Line 1: Six in ten will not be calculated as 42.3%. Please check this and it may be 60%.

Response: We note that the combination of disagree and strongly disagree totals 63%. Sixty-three percent of women chose one of these two options. We have included "either" to further clarify this point.

Line 1: If disagreed and strongly disagreed are different "variables", then "or" should be replaced with "and".

Response: As above, we have included either to further clarify this point.

Discussion: Please replace discussion with a conclusion. Also check ".........other mass media campaigns are necessary are critical,". Did the authors want to say, "necessary and critical".

Review: Per Reviewer 1 comments, the abstracts conclusions have been rewritten and this sentence was removed.

Strengths and limitations of the study

Comment: Please add a sentence or two about what new knowledge the study adds to existing knowledge and available literature. Then reduce the length of this section.

Response: Thank you for this suggestion, however, we followed the instructions to authors for BMJ open which state "Please include a 'Strengths and limitations of this study' section after the abstract. This section should be no more than 5 bullet points relating specifically to the methods - not the results of the study. This will be published as a summary box after the abstract in the final published article."

Main document

Background:

Comment 1: Line 75 - 79...Consider reducing the length of sentences. That gives a better meaning to readers.

Response: We have modified the sentence into two separate sentences.

Modified text (lines 71-75): Concerns about contraceptive-induced fertility impairment, whether delayed fertility or permanent infertility, are so influential that they may preclude use of hormonal contraception altogether. Instead, women, particularly young women, may rely on either abortion (11) or less effective methods of contraception to regulate unwanted fertility (12).

Comment 2: Authors may want to explain what they mean by " ...... amplified when network members also held this belief...."

Response: We have modified the sentence to clarify.

Modified text (lines87-90): Social network research in Kenya found that the individual belief that contraception can cause infertility was associated with lower contraceptive use, and that when members of an individual's network also held this belief, contraceptive use was further reduced (17).

Comment 3: line 90: ...... strongly agreed that the use of contraception...."

Response: We have added the definite article as suggested.

Line 91 – 92: ...... were significantly less likely to adopt contraception over a one-year period; there was no association, however, between levels of community agreement and uptake". Authors may want to explain this better. The two sentences really are not connected.

Response: We have modified the sentence to clarify.

Modified text (lines 90-94): Longitudinal evidence from Uganda found that women who strongly agreed that the use of contraception made it harder to get pregnant later were significantly less likely to adopt contraception over a one-year period than women who did not hold this belief. There was no association, however, between higher levels of agreement with this belief in the community and an individual's adoption of contraception (18).

Comment 4, line 96 - 97: that results differed between urban and rural settings speak to the diverse ways in which this concern and the social environment can influence contraceptive use. Please rephrase this sentence clearly.

Response: We have modified the sentence for greater clarity.

Modified text (line 96-99): Though Odwe and colleagues did not specifically explore community influences on use, that results differed between urban and rural settings underscores that the influence of the social environment on contraceptive use is not uniform and can vary based on location and setting (19).

General comment: Authors may want to consider adding more data to demonstrate broader evidence of the subject in Sub-Saharan Africa. Also compare statistical evidence between Ethiopia and other African countries, not just Kanya. Ensure that sentences and paragraphs are connected in meaning and content.

Response: Thank you for this comment. We agree that it would be extremely valuable to reference data specific to Ethiopia, however, there are limited studies, other than those mentioned, that specifically explore concerns about contraceptive induced infertility. As mentioned, while there is substantial qualitative data that supports the importance of this question, quantitative research is limited. We have included the limited quantitative studies that were available and believe the general lack of high-quality quantitative research on this subject further justifies the publication of this manuscript.

Methods, Data:

Comment 1,: Please state specifically the multistage sampling method that was used by PMA or give a reference for readers.

Response: We have included an additional citation to the PMA Ethiopia protocol, which is included at the end of the paragraph. Additional detail on survey design, including detailed sampling information, is provided therein.

Comment 2: Multilevel hierarchical models assessed how agreement is associated with the use of a hormonal method of contraception among 3,882 and descriptive statistics used among a sample of 7,491 women. In total, 7,533 de facto women completed the survey (refer to main body)? Can authors explain if the 3,882 sample is part of the 7,491 women? Refer to abstract and methods in main document. Also reconcile the 7,533 sample with the rest. Please make clear as it is under the heading “Analytic sample” but reconcile with 7,533 sample.

Response: We have attempted to further clarify that the analytic sample of 3,882 arose from the original 7,533 women with additional detail included at line 238 and line 245.

Include a sentence about ethical consideration for women less than 18 years of age. Were they also under oral consent and by parents or not?

Response: We have provided additional detail that per the National Research Ethics Review Guidelines and the IRB at AAU, women age 15-17 are able to consent from themselves and parental consent was not required.

Additional language (lines 158-160): Additionally, women age 15-17 are considered able to consent from themselves for studies that cover sensitive topics, including reproductive health, and parental consent was not required per the National Research Ethics Review Guidelines.

Outcome Measures:

Comment 1: How did authors measure the second outcome “current use of a hormonal method of contraception”?

Response: Additional detail was added to clarify what we considered current use of a method, in addition to further detail requested by Reviewer 1.

Modified/additional text (line 194-198): Women were first asked whether they had heard of each method of contraception, including lactational amenorrhea and traditional methods, and then asked if they or their partner were doing anything to prevent pregnancy. If so, they were asked to specify which method or methods they were using and were considered current users if they reported using any of the above-mentioned methods.

Line 190: Was “reported infertility” viewed by authors as self-reported or considered as contraceptive-induced?

Response: “Reported infertile” is in response to a question on how long a woman would like to wait before becoming pregnant. Women may report that they are not able to become pregnant and are thus considered infertile by self-report. There are no additional questions in the questionnaire to clarify why a woman may consider herself to be infertile. We have clarified that this is considered self-report at line 208.

Line 197 -198: If authors categorized “neither agreed nor disagreed” as agree, was this analyzed as a separate variable or combined with “strongly agreed or agreed” as a single variable? I would have thought that this would be an independent/neutral group. Authors previously stated in line 175 that “respondents who were ambivalent were categorized as “neither agree nor disagree”, then further categorize this as agree.

Response: We note that at lines 213-217, fear of contraceptive-induced fertility impairment was coded as a three-level categorical variable, in which strongly agree, agree, and neither agree nor disagree were considered one category, disagree was a second, and strongly disagree was a third. As noted, there were no meaningful differences between strongly agree, agree, and neither agree nor disagree when treated as separate categories within the categorical variable. To maximize statistical power, these were thus combined into a single category. We note that we did not reclassify women from ambivalent to “neither agree nor disagree”, but that interviewers were trained to select “neither agree nor disagree” if women were ambivalent in their response.

Define your terms as they relate to your study variables for a wider audience. E.g: contraceptive-induced infertility, multilevel hierarchical models, parity nulliparous and primiparous, etc.

Response: We have attempted to simplify the language throughout and appreciate the reviewers' commitment to making research understandable to a wide audience. However, some statistical language was left as originally written.

Results

Comment 1: Authors may want to explain (in a single sentence) the concept of full sample and analytic sample in the methods section before incorporating it here.

Response: We note that there is a specific section entitled “Analytic sample” which describes both the full and reduced sample and the rationale for using both samples. We hope that this is sufficient but if the editors believe further detail is necessary, we are happy to make further edits.

Line 261-262: 40% is less than the other half (60%) and cannot be considered as the larger percentage.

Response: We have attempted to clarify that the response option which garnered the most responses was “disagreed”, at 40%.

Modified text (line 289-291): There are significant differences across all sociodemographic characteristics, however, across all background characteristics more women stated that they disagreed with the statement—roughly 40%— than any other option.

Line 292: ".... among women who live in a community where no women agreed...". Did authors want to mean no woman or no women?

Response: We believe that this is correct as written, but will be pleased to modify if required.

Authors should give brief descriptions of figures.

Response: Thank you. We note that Figure 1 is summarized at line 287 while Figure 2 is summarized at line 327, noting that Figure 2 is a visual demonstration of the previously presented results.

General comment: Please move all table headings to where tables are located or delete table headings from results section if tables below already contain headings. Only reference text descriptions in the results corresponding to tables.

Response: We appreciate the reviewer's suggestion, but have left the table and figure titles within the text per guidance provided by the journal. We defer to the editor as to whether any additional formatting changes are required and will be pleased to do them.

Discussion

Comment 1, line 312: please refer to comment on the abstract.

Response: As noted above, six in ten women either disagreed or strongly disagreed. The cumulative total of these two options was 63%.

Comment 1: Authors may want to reduce the length of sentences to make meaning.

Response: Thank you. We have attempted to simplify language throughout.

Comment 1: Authors may want to compare results to more findings than they already did. More is needed on which other findings by other authors are different and which are like current findings?

Response: Thank you. Based on this and the other reviewer's comments, we have made significant changes throughout.

References

Comment 1: Authors may want to review old references and update with recent ones.

Response: Thank you, however there are limited quantitative studies which specifically explore this question. We attempted to include the most relevant and recent research on the subject.

VERSION 2 – REVIEW

**REVIEWER NAME**	***Rose Stevens***
**REVIEWER AFFILIATION**	University of Oxford Social Sciences Division
**REVIEWER CONFLICT OF INTEREST**	None

DATE REVIEW RETURNED	15-Dec-2023

GENERAL COMMENTS	I would like to thank the authors for the extensive additions and revisions made to the manuscript. I am satisfied that all the reviews are addressed or responded to. I think this paper is a very strong contribution to the literature from a unique data source with a novel and interesting analysis. I happily recommend its publication.

VERSION 3 – REVIEW

REVIEWER NAME	***Elvis Junior Dun-Dery***
REVIEWER AFFILIATION	Oregon State University, Public health and Nutrition
REVIEWER CONFLICT OF INTEREST	I have no competing interest
DATE REVIEW RETURNED	12-Apr-2024

GENERAL COMMENTS	Review report.<br>Assessing the effect of concerns about contraceptive induced fertility impairment on hormonal contraceptive use by parity and residence; evidence from PMA Ethiopia 2020 cross-sectional survey.<br><br>Thank you for the opportunity to review this manuscript again. The authors made some changes. However, below are my comments to consider in improving the standard of the paper.<br><br>Abstract<br>There should be a column after each heading. That is "Objectives: "<br>Line 30: "We used multilevel hierarchical…."<br>Line 33: Six in ten is not 42.3%. Authors may want to rework this.<br>Line 36: What effect was strongest….? Specify the relationship here.<br>Line 37: Greater agreement with what statement? If it's the statement "contraception leading to infertility", please specify that.<br>The heading "discussion" should be changed to conclusion.<br><br>Background<br>Line 67: "Fears regarding contraceptive-induced…." Can not go with "…a critical barrier..." Authors should correct the grammar.<br>Authors should attach references to specific statements. Readers should be able to connect ideas to specific authors and references in the reference list. Giving a range of references for each sentence is not helpful.<br>Lines 68 to 72: This body of evidence sounds contradictory and needs to be reconciled. How can available evidence support both opposing views of no long-term effects and effects exceeding one year? Which one is the focus of the authors and why discuss both opposing evidence? For instance, "Existing evidence indicates no long-term impacts of contraceptive use on fecundity………..up to and exceeding one year".<br>Line 76 to 77: What do you mean by "unwanted fertility"?. Many readers would justify unwanted pregnancy but how is unwanted fertility regulated? I am sure authors are refereeing to births that were not wanted at the time of conception, which is actually "unwanted fertility. Please define that here.<br>Lines 77 to 79: There is no clarity here. Abortion is not a hormonal contraception, and the authors did not specify the alternative

	methods used by women to regulate fertility here. So, they can't subsequently discuss how using alternatives is not problematic. Additionally, authors moved straight to discussing decision making using inaccurate or incomplete information and autonomy. These concepts were not previously introduced, and the authors did not create a link between limited information or autonomy to contraception or infertility due to contraception. So, a sudden introduction is out of place.<br>Line 80: What do you mean by "high and low fertility settings" ? Specify, if continental difference is the focus, else, justify what factors should be in place to consider a place "low or high fertility setting".<br>Line 81: Your study is not a qualitative study. Refer to more quantitative studies.<br>Line 97 to 100: This sentence does not connect grammatically. "….that results differed between urban and rural settings underscores that the influence of the social….."<br>Line 104: The influence of what belief? Be specific if you are referring to contraceptives causing infertility.<br>Line 109: Evidence suggests that not "Evidence has suggested" . Fear of infertility not "fears" .<br>Line 113: Almost half here is what percentage of the study sample? Indicate how many in percentages. "Ethiopian female, rural, married, non-users of contraception, almost half believed that contraception."<br>Lines 114 to 115: Rewrite this sentence. "…..given the pressure women are under" is not scholarly language.<br>Line 116: Unable to explore which question? If your current research question, please be specific.<br>Authors have not discussed in detail the community level beliefs of contraceptive-induced infertility. This is a major objective, as indicated by the authors, yet discussions on this are limited in the background.<br>Line 133 to 136: "….the wording of the question, described in detail below……" This language is not scholarly. Authors may want to rewrite this section to convey their ide in a better way.<br><br>Methods<br>Data<br>Lines 163 to 166: From the research narrative and source of data, the researchers used secondary data. How was this study ethically approved by the Johns Jopkins University and the University of Addis Ababa? Ethical reference should be made to the original ethics committee/board that reviewed the protocol before the study was conducted. You do not need a second ethical clearance to use secondary data, but you still need to reference the initial ethics board and clearly state the procedures that were used.<br>Outcome<br>The authors will need to reconcile how they translated the study outcome from the entire SSA to an outcome in Ethiopia and applied that to an already existing dataset (the PMA). The only aspect I can see transferable is how the outcome was measured, which is what the authors did here, but how did you modify same outcome to fit into your data, considering that different variables were measured in the two studies? Also tell readers how the variable of interest ("If I use family planning, I may have trouble getting pregnant next time I want to") was measured in the PMA data and why that was not used for the analysis but rather, the outcome from the WGE-SRH study.<br>Lines 184 to 186: The term family planning may be widely known to be synonymous with modern contraception but not technically correct. Did your study seek to measure FP or modern

	contraception? You should be defining this and narrowing your focus to which one was measured in your study. Also reference the studies with the findings indicating FP as synonymous to modern contraception. Qualitative studies and pilot studies cannot qualify definitions to be used in your study. Qualitative studies will not tell us how many women thinks FP is synonymous to MC. Numbers matter in generalizing concepts and making conclusions on theories.<br>Lines 199 to 202: Combining condom and traditional method users with non-users will not be correct here. People who use condoms are different from those who use other traditional methods, and the two groups are also different from those who don't use any contraceptives method at all. These groups cannot be added together. You need to separate these groups and refit your models. Group difference is important in understanding effect size on the outcome you are measuring.<br>Explanatory variables<br>Lines 209 to 211: Did the authors detect any collinearity between household items and household construction? Why conduct principal component analysis here with these two variables? Also note that the DHS methods are slightly different from those used in the PMA and WGE-SRH data collection methods. Transcend between these data sets with caution.<br>Lines 217 to 218: Use dose response relationship here cautiously. There are no hazard measures/effects or drug tests here. You are simply measuring how participants agree or disagree with the outcome.<br>Lines 218 to 219: I am sure readers will have a challenge with how you are using simplicity to combine variables for measurements and model development. "Strongly agreed, agreed, or neither agreed nor disagreed as "agree" are different levels of acceptance and cannot be combined.<br>Lines 221: "…..either that they neither agreed nor disagreed……"Grammatically incorrect.<br><br>Analysis<br>Describe which survey weights were used here. How did you calculate the weight before applying it to the data and was this applied in all levels of analysis or just descriptive statistics? There is a formular for calculation survey weight and that should be made clear here.<br><br>Results<br>Line 302: significantly negative not "significant negative". Check this throughout the results.<br>Line 308: this figure should be 26% not 25%<br>Line 331: :…. previous model at .250 (not shown). What is the .250 here?<br>General comment: I did not review the entire results because of previous recommendation to rework parts of the results.<br><br><br>Discussion<br>Lines 349: Are the authors referring to this part of the table as indicating 6 in 10 women either disagreed or strongly disagreed?<br>I do not see any figure representing 6 in 10 women here.<br>Lines 353 to 357: This section is not needed. Restating your hypothesis in the discussion is not necessary. Instead, you should be comparing your results to findings from the studies you cited.<br>General comment: Apart from the last few sentences, I do not see a lot of comparison with other studies. Readers need to know which

	other studies support your findings or have contrary findings. A lot of the sections in the discussion are repeats of the results without statistics. Authors may want to review this and rework it.

VERSION 3 – AUTHOR RESPONSE

Reviewer: 2
Dr. Elvis Junior Dun-Dery, Oregon State University
Comments to the Author:
Review report.
Assessing the effect of concerns about contraceptive induced fertility impairment on hormonal contraceptive use by parity and residence; evidence from PMA Ethiopia 2020 cross-sectional survey.

Thank you for the opportunity to review this manuscript again. The authors made some changes. However, below are my comments to consider in improving the standard of the paper.

Abstract
There should be a column after each heading. That is “Objectives: “

Response: Thank you for the comment. We note that in published abstracts, BMJ Open does not include colons. We have cited a handful of recently published papers showing this, however, we are happy to defer to the editor’s decision.

Riku Y, Atsuta N, Yoshida M*, et al*. Differential motor neuron involvement in progressive muscular atrophy: a comparative study with amyotrophic lateral sclerosis. *BMJ Open* 2014;4:e005213. doi: 10.1136/bmjopen-2014-005213

Tumlinson K, Senderowicz L, Bullington BW*, et al*. Assessing trends and reasons for unsuccessful implant discontinuation in Burkina Faso and Kenya between 2016 and 2020: a cross-sectional study. *BMJ Open* 2023;13:e071775. doi: 10.1136/bmjopen-2023-071775

Footman K. Interviewer effects on abortion reporting: a multilevel analysis of household survey responses in Côte d’Ivoire, Nigeria and Rajasthan, India. *BMJ Open* 2021;11:e047570. doi: 10.1136/bmjopen-2020-047570

Line 30: “We used multilevel hierarchical….”

Response: Thank you. This has been addressed.

Line 33: Six in ten is not 42.3%. Authors may want to rework this.

Response: Thank you for the comment. To clarify, we now note that approximately 4 in ten women disagreed (42.3%) and 2 in ten strongly disagreed (20.7%) for a total of 6 in ten stating either that they disagreed or strongly disagreed. We have revised the abstract for further clarity.

Line 36: What effect was strongest….? Specify the relationship here.

Response: We have further clarified that the effect of agreeing with the statement was strongest amongst high parity women.

Line 37: Greater agreement with what statement? If it's the statement "contraception leading to infertility", please specify that.

Response: Given word limits in the abstract, we felt that we did not have sufficient room to repeat the wording of the statement beyond its original inclusion. We defer to the editor as to whether word limits can be exceeded.

The heading "discussion" should be changed to conclusion.

Response: Thank you. We have made this change.

Background
Line 67: "Fears regarding contraceptive-induced…." Can not go with "…a critical barrier..." Authors should correct the grammar.

Response: This has been corrected.

Authors should attach references to specific statements. Readers should be able to connect ideas to specific authors and references in the reference list. Giving a range of references for each sentence is not helpful.

Response: We note that multiple citations are used when several studies have reached similar conclusions. We provide several recently published papers from BMJ Open that have employed similar referencing approaches.

Zemedikun DT, Chandan JS, Raindi D*, et al*. Burden of chronic diseases associated with periodontal diseases: a retrospective cohort study using UK primary care data *BMJ Open* 2021;11:e048296. doi: 10.1136/bmjopen-2020-048296

Bjornsdottir E, Thorarinsdottir EH, Lindberg E*, et al*. Association between physical activity over a 10-year period and current insomnia symptoms, sleep duration and daytime sleepiness: a European population-based study. *BMJ Open* 2024;14:e067197. doi: 10.1136/bmjopen-2022-067197

Moreau C, Karp C, Wood S*, et al*. Trends in fertility intentions and contraceptive practices in the context of COVID-19 in sub-Saharan Africa: insights from four national and regional population-based cohorts. *BMJ Open* 2023;13:e062385. doi: 10.1136/bmjopen-2022-062385

Lines 68 to 72: This body of evidence sounds contradictory and needs to be reconciled. How can available evidence support both opposing views of no long-term effects and effects exceeding one year? Which one is the focus of the authors and why discuss both opposing evidence? For instance, "Existing evidence indicates no long-term impacts of contraceptive use on fecundity………..up to and exceeding one year".

Response: We have attempted to clarify that there are no permanent effects on fecundity from the use of hormonal contraception, however there is evidence that discontinuation of injectable methods of contraception can lead to sub-fecundity for up to a year. We note that this clarification was requested by the other reviewer. The updated text now reads "Existing evidence indicates no permanent impacts of contraceptive use on fecundity following discontinuation. Research does, however, suggests delays in return to fecundity for users of some hormonal methods – mainly injectable contraceptives - with delays of a subsequent pregnancy following discontinuation up to and exceeding one year (9,10). "

Line 76 to 77: What do you mean by "unwanted fertility"?. Many readers would justify unwanted pregnancy but how is unwanted fertility regulated? I am sure authors are refereeing to births that were not wanted at the time of conception, which is actually "unwanted fertility. Please define that here.

Response: We thank the reviewer for raising this important question. Women may use contraceptive methods to prevent an unwanted pregnancy and/or use abortion to prevent an unwanted birth. As abortion cannot be used to prevent unwanted pregnancy, but can only be used to prevent an unwanted birth once pregnant, we did not think that use of the term "unwanted pregnancy" was appropriate in this sentence.

Lines 77 to 79: There is no clarity here. Abortion is not a hormonal contraception, and the authors did not specify the alternative methods used by women to regulate fertility here. So, they can't subsequently discuss how using alternatives is not problematic.

Response: We have attempted to clarify what we mean by "alternative methods of contraception" (non-hormonal), specifically traditional methods and condoms.

Additionally, authors moved straight to discussing decision making using inaccurate or incomplete information and autonomy. These concepts were not previously introduced, and the authors did not

create a link between limited information or autonomy to contraception or infertility due to contraception. So, a sudden introduction is out of place.

Response: Thank you for the note. We believe that including these concepts is appropriate given the importance of a woman's individual autonomy on decision-making. We defer to the editor on whether the introduction of informed decision-making and autonomy should be removed.

Line 80: What do you mean by "high and low fertility settings" ? Specify, if continental difference is the focus, else, justify what factors should be in place to consider a place "low or high fertility setting".

Response: We appreciate this comment. We have modified the language to reflect that this finding has been found across "diverse settings", rather than focusing on high versus low fertility levels specifically, as there is no standard definition of what is considered high versus low.

Line 81: Your study is not a qualitative study. Refer to more quantitative studies.

Response: Thank you for the comment. Qualitative research has, until recently, formed the majority of the research base related to concerns around contraceptive induced fertility impairment. We believe that citing this is critical as it underscores that it is an important concern that has largely not been explored quantitatively.

Line 97 to 100: This sentence does not connect grammatically. "….that results differed between urban and rural settings underscores that the influence of the social….."

Response: We have modified the sentence, which now reads: "Though Odwe and colleagues did not specifically explore community influences on use, the fact that results differed between urban and rural settings underscores that the influence of the social environment on contraceptive use is not uniform and can vary based on location and setting (19)."

Line 104: The influence of what belief? Be specific if you are referring to contraceptives causing infertility.

Response: We have modified the sentence, which now reads "While studies suggest that the influence of fears regarding contraceptive-induced fertility impairment may vary based on environment, such as urban and rural residence (19,20), none have specifically explored whether the influence of contraceptive-induced fertility impairment might also vary over the life course, such as with increased age or parity."

Line 109: Evidence suggests that not "Evidence has suggested" . Fear of infertility not "fears" .

Response: We have modified the sentence.

Line 113: Almost half here is what percentage of the study sample? Indicate how many in percentages. “Ethiopian female, rural, married, non-users of contraception, almost half believed that contraception.”

Response: We have modified the sentence. The updated sentence now reads “Sedlander and colleagues recently found that among Ethiopian female, rural, married, non-users of contraception, almost half (48.2%) believed that contraception caused infertility (20).”

Lines 114 to 115: Rewrite this sentence. “…..given the pressure women are under” is not scholarly language.

Response: We clarified that we mean social and familial pressure in the text, however, we also note that multiple scholarly papers have used language related to societal pressure to bear children, including the originally referenced paper, and have cited a small sample of these below. Such pressure includes the internal and external factors that shape women’s decision-making related to reproductive decisions. The updated text now reads “They note that the importance of this concern is likely to vary by parity, given the social and familial pressures that women are under to demonstrate fertility upon marriage (20,23), but were unable to explore how concerns about contraceptive-induced fertility impairment varied by parity.”

Sekine K, Khadka N, Carandang RR, et alMultilevel factors influencing contraceptive use and childbearing among adolescent girls in Bara district of Nepal: a qualitative study using the socioecological modelBMJ Open 2021;11:e046156. doi: 10.1136/bmjopen-2020-046156

Yu, J., Grace, K., Boyle, E.H., Mikal, J.P., Gunther, M. and Kristiansen, D. (2024), COVID-19 and Contraceptive Use in Two African Countries: Examining Conflicting Pressures on Women. Population and Development Review. https://doi.org/10.1111/padr.12606

Odoi EY. Experiences of pressure to have children among mothers in Accra, Ghana. Afr J Reprod Health. 2023 Dec 31;27(12):43-50. doi: 10.29063/ajrh2023/v27i12.5. PMID: 38158861.

Line 116: Unable to explore which question? If your current research question, please be specific.

Response: We have modified the sentence. The updated text now reads “The updated text now reads “They note that the importance of this concern is likely to vary by parity, given the

social and familial pressures that women are under to demonstrate fertility upon marriage (20,23), but were unable to explore how concerns about contraceptive-induced fertility impairment varied by parity."

Authors have not discussed in detail the community level beliefs of contraceptive-induced infertility. This is a major objective, as indicated by the authors, yet discussions on this are limited in the background.

Response: Thank you for this comment. We clarify that the second paragraph includes discussion of the role of community level beliefs. We note that there are no other papers that we are aware of that have quantitatively explored the association levels of community agreement with this belief (i.e. fear of contraceptive induced fertility impairment) and individual contraceptive behavior.

Line 133 to 136: "….the wording of the question, described in detail below……" This language is not scholarly. Authors may want to rewrite this section to convey their ide in a better way.

Response: We note that this clarification was included at the request of the other reviewer, who did not note any specific concerns with this language. Given that this was already approved by the other reviewer, we prefer to leave the language as written, but will defer to the editor.

Methods
Data
Lines 163 to 166: From the research narrative and source of data, the researchers used secondary data. How was this study ethically approved by the Johns Hopkins University and the University of Addis Ababa? Ethical reference should be made to the original ethics committee/board that reviewed the protocol before the study was conducted. You do not need a second ethical clearance to use secondary data, but you still need to reference the initial ethics board and clearly state the procedures that were used.

Response: All authors, with the exception of one, are members of the PMA Ethiopia study team. We did not consider this a secondary analysis as it was written by study team members, during the grant period. As such, the details that are provided are those of the original PMA Ethiopia ethics approval.

Outcome
The authors will need to reconcile how they translated the study outcome from the entire SSA to an outcome in Ethiopia and applied that to an already existing dataset (the PMA). The only aspect I can see transferable is how the outcome was measured, which is what the authors did here, but how did you modify same outcome to fit into your data, considering that different variables were measured in the two studies? Also tell readers how the variable of interest ("If I use family planning, I may have

trouble getting pregnant next time I want to") was measured in the PMA data and why that was not used for the analysis but rather, the outcome from the WGE-SRH study.

Response: We appreciate the reviewer's question. We clarify that the survey item was originally developed from formative research across multiple countries, including Ethiopia, as part of the broader PMA project. The statement of interest, "If I use family planning, I may have trouble getting pregnant the next time I want to", was developed from the formative research process for the Women's and Girls' Empowerment in Sexual and Reproductive Health (WGE-SRH) project, which included qualitative research to identify women's internalized fears and external motivations for reproductive health decisions. This item, among the others developed from qualitative work, was further tested in quantitative surveys, and used, without modification, in the PMA Ethiopia survey. Cognitive testing was done prior to inclusion in PMA Ethiopia to ensure that the question was understood by participants. The WGE-SRH survey included a range of additional questions and outcomes, which are outside the scope of this paper. We included this level of detail to clarify how the item was originally constructed for the reader's knowledge. For this analysis, we focus exclusively on women's response to this single item as a reflection of their concerns about contraceptive-induced fertility impairment specifically.

Lines 184 to 186: The term family planning may be widely known to be synonymous with modern contraception but not technically correct. Did your study seek to measure FP or modern contraception? You should be defining this and narrowing your focus to which one was measured in your study. Also reference the studies with the findings indicating FP as synonymous to modern contraception. Qualitative studies and pilot studies cannot qualify definitions to be used in your study. Qualitative studies will not tell us how many women thinks FP is synonymous to MC. Numbers matter in generalizing concepts and making conclusions on theories.

Response: We thank the reviewer for this comment. We agree that there is a difference between contraception and family planning, particularly in the academic and scientific fields, where family planning can refer to a broader range of methods and behaviors that prevent birth, including abortion. However, as noted, during cognitive interviews, our respondents in Ethiopia did not make this academic or scientific distinction and rather use the terms family planning interchangeably with contraception. Indeed, the words are translated the same across the local languages. We feel it is critical to use the terms and language used by respondents to the PMA Ethiopia survey. We did not publish on the specific cognitive interview results and thus cannot reference a specific paper that supports this assertion. We agree that greater specification in wording will improve scientific processes, however, also note that it is important to use the words and concepts of a population when developing survey items.

Lines 199 to 202: Combining condom and traditional method users with non-users will not be correct here. People who use condoms are different from those who use other traditional methods, and the two groups are also different from those who don't use any contraceptives method at all. These groups cannot be added together. You need to separate these groups and refit your models. Group difference is important in understanding effect size on the outcome you are measuring.

Response: We agree that condom users may differ from traditional method users and non-users of any method of contraception. If there was a sufficient sample size available for meaningful interpretation, we agree it would be helpful to separate these groups for analysis. However, with only 13 condom users and 127 traditional method users, we do not believe that there is sufficient sample to draw meaningful conclusions. If anything, the inclusion of these small sub-populations as currently done is likely to lead to conservative results. We appreciate the reviewer's suggestion, but note that these details were included at the request of the other reviewer who did not raise similar concerns. We also note that traditional method users are commonly combined with non-users of contraception when attempting to identify meaningful influences on use and non-use of a modern contraceptive method specifically. We have included papers below that have used such definitions in Ethiopia.

Zeleke, G.T., Zemedu, T.G. Modern contraception utilization and associated factors among all women aged 15–49 in Ethiopia: evidence from the 2019 Ethiopian Mini Demographic and Health Survey. *BMC Women's Health* 23, 51 (2023). https://doi.org/10.1186/s12905-023-02203-8

Belachew TB, Negash WD, Bitew DA, et alModern contraceptive utilisation and its associated factors among reproductive age women in high fertility regions of Ethiopia: a multilevel analysis of Ethiopia Demographic and Health SurveyBMJ Open 2023;13:e066432. doi: 10.1136/bmjopen-2022-066432

Tsehay CTFactors associated with modern contraceptive demands satisfied among currently married/in-union women of reproductive age in Ethiopia: a multilevel analysis of the 2016 Demographic and Health SurveyBMJ Open 2022;12:e049341. doi: 10.1136/bmjopen-2021-049341

Explanatory variables
Lines 209 to 211: Did the authors detect any collinearity between household items and household construction? Why conduct principal component analysis here with these two variables? Also note that the DHS methods are slightly different from those used in the PMA and WGE-SRH data collection methods. Transcend between these data sets with caution.

Response: We note that we did not conduct a separate analysis for this paper to estimate the wealth index but rather used the index that is generated as part of the standard PMA Ethiopia data analysis protocol and which is cited in the previously published protocol paper. We, thus, did not identify any collinearity in these specific variables nor conduct principal components analysis as part of this analysis. We note, however, that the PMA Ethiopia questions that are included in the survey are based off of those in the DHS-7 (DHS version when the PMA Ethiopia questionnaires were originally developed), including using the same questions for household infrastructure and assets. The analysis that is employed to construct the wealth quintiles uses the same questions and analytic methods as the DHS.

Lines 217 to 218: Use dose response relationship here cautiously. There are no hazard measures/effects or drug tests here. You are simply measuring how participants agree or disagree with the outcome.

Response: We have modified the sentence accordingly. The updated text now reads "After exploratory analyses, we coded fear of contraceptive-induced fertility impairment into a three-level categorical variable, indicating 1) strongly agree/agree/neither agree nor disagree, 2) disagree, and 3) strongly disagree as the relationship with use did not vary by strongly agree, agree, and neither agree nor disagree but demonstrated a clear increase in association thereafter (Supplemental Figure 1)."

Lines 218 to 219: I am sure readers will have a challenge with how you are using simplicity to combine variables for measurements and model development. "Strongly agreed, agreed, or neither agreed nor disagreed as "agree" are different levels of acceptance and cannot be combined.

Response: We thank the reviewer for this point. While we agree that these may differ conceptually, the lack of meaningful difference in their effect size did not justify their inclusion as separate variables. As such, we chose to combine in order to maximize the sample size and statistical power. Without this combination, our power was limited and we could not draw meaningful statistical inference. As with the combination of condom and traditional method users described above, the combination of these responses is likely to have led to conservative estimates of the effect of disagreeing or strongly disagreeing with the statement about contraceptive-induced fertility impairment. Additionally, women who endorse this statement, whether they express "strong agreement" or "agreement", demonstrate that they hold concerns about how contraceptive practices may affect future fertility; this is distinct from individuals who do not endorse this belief for whom contraception is not seen as a potential impairment for fertility. For this reason, we feel that pooling these response categories as "endorsement" of this belief is appropriate for the current analysis.

Lines 221: "…..either that they neither agreed nor disagreed……"Grammatically incorrect.

Response: We have revised the sentence.

Analysis

Describe which survey weights were used here. How did you calculate the weight before applying it to the data and was this applied in all levels of analysis or just descriptive statistics? There is a formular for calculation survey weight and that should be made clear here.

Response: We specify in line 365-369 that descriptive analyses utilized survey weights and have noted that additional detail on the construction of survey weights is available in the PMA Ethiopia survey protocol. For the sake of length, we did not repeat detail on construction of survey weights here. We have linked to other papers published in BMJ Open that have provided similar detail when discussing the utilization of survey weights.

Moreau C, Karp C, Wood S, Williams K, Olaolorun FM, Akilimali P, Guiella G, Gichangi P, Zimmerman L, Anglewicz P. Trends in fertility intentions and contraceptive practices in the context of COVID-19 in sub-Saharan Africa: insights from four national and regional population-based cohorts. BMJ Open. 2023 Jan 18;13(1):e062385. doi: 10.1136/bmjopen-2022-062385. PMID: 36657770; PMCID: PMC9852736.

Tumlinson K, Senderowicz L, Bullington BW, et alAssessing trends and reasons for unsuccessful implant discontinuation in Burkina Faso and Kenya between 2016 and 2020: a cross-sectional study. BMJ Open 2023;13:e071775. doi: 10.1136/bmjopen-2023-071775

Footman K. Interviewer effects on abortion reporting: a multilevel analysis of household survey responses in Côte d'Ivoire, Nigeria and Rajasthan, India. BMJ Open 2021;11:e047570. doi: 10.1136/bmjopen-2020-047570

Results

Line 302: significantly negative not "significant negative". Check this throughout the results.

Response: We believe that our grammar is correct, but defer to the editor.

Line 308: this figure should be 26% not 25%

Response: We have modified the language to clarify that approximately one-quarter of variation was due to variation across clusters.

Line 331: :…. previous model at .250 (not shown). What is the .250 here?

Response: This refers to the Intraclass Correlation, estimating the percentage of variation in contraceptive use that was due to variation across clusters. This was previously requested by the other reviewer.

General comment: I did not review the entire results because of previous recommendation to rework parts of the results.

Response: We thank the reviewer for their previous comments.

Discussion

Lines 349: Are the authors referring to this part of the table as indicating 6 in 10 women either disagreed or strongly disagreed? I do not see any figure representing 6 in 10 women here.

Response: We have attempted to provide further clarification, noting that approximately 40% of women disagreed and 20% strongly disagreed, totaling sixty percent or six in ten.

Lines 353 to 357: This section is not needed. Restating your hypothesis in the discussion is not necessary. Instead, you should be comparing your results to findings from the studies you cited.

Response: We have modified the discussion so that our findings are more clearly situated within the limited research base that quantitatively explores the influence of fears surrounding contraceptive-induced fertility impairment.

General comment: Apart from the last few sentences, I do not see a lot of comparison with other studies. Readers need to know which other studies support your findings or have contrary findings. A lot of the sections in the discussion are repeats of the results without statistics. Authors may want to review this and rework it.

Response: We have modified the discussion to better align with other studies and have noted the dearth of quantitative studies that have specifically explored this question, thus limiting our ability to compare to other studies.

VERSION 4 – REVIEW

**REVIEWER NAME**	***Elvis Junior Dun-Dery***
**REVIEWER AFFILIATION**	Oregon State University, Public health and Nutrition
**REVIEWER CONFLICT OF INTEREST**	I have no competing interest
**DATE REVIEW RETURNED**	14-Jun-2024

**GENERAL COMMENTS**	Review report – BMJ Open<br>Assessing the effect of concerns about contraceptive induced fertility impairment on hormonal contraceptive use by parity and residence; evidence from PMA Ethiopia 2020 cross-sectional survey.<br><br>Line 101: Contraceptives use was further….<br>Line 105: “higher levels of community agreement with this belief”. Specify the belief here.<br><br>Tables:<br>Rework tables to fit publishable standard. Remove boarders and refer to other published work to ensure this is done.<br>All p-values that are written on tables as 0.00 should be changed to 0.001. Ensure this is done before resubmitting. Maybe I missed this, but it was part of my previous review comments.

VERSION 4 – AUTHOR RESPONSE

We thank the reviewer for their careful review of our manuscript. Please see our response to the minor revisions suggested by the reviewer. In addition, please note that a query was sent to the editors on July 23 requesting for any further clarification on modifications to the tables that are necessary to meet publishable standards. We will be pleased to make any further revisions as required by the journal.

Line 101: Contraceptives use was further….

Response: We cannot find the exact line that the reviewer is referencing in either the clean or track change version, however, we note that the correct language is “contraceptive use” not “contraceptives use” and there is no reference to “contraceptives use” in the manuscript.

Line 105: “higher levels of community agreement with this belief”. Specify the belief here.

Response: We have modified the sentence as follows: “There was no association, however, between higher levels of agreement within the community that contraception made it harder to get pregnant and an individual’s adoption of contraception (18).”

Tables: Rework tables to fit publishable standard. Remove boarders and refer to other published work to ensure this is done. All p-values that are written on tables as 0.00 should be changed to 0.001. Ensure this is done before resubmitting. Maybe I missed this, but it was part of my previous review comments

Response: We have modified the tables to remove the internal borders and updated the p-values as recommended. We thank the reviewer for their careful review.
